# Accelerating the Development of Heat Tolerant Tomato Hybrids through a Multi-Traits Evaluation of Parental Lines Combining Phenotypic and Genotypic Analysis

**DOI:** 10.3390/plants10102168

**Published:** 2021-10-13

**Authors:** Fabrizio Olivieri, Salvatore Graci, Silvana Francesca, Maria Manuela Rigano, Amalia Barone

**Affiliations:** Department of Agricultural Sciences, University of Naples Federico II, Via Università 100, 80055 Portici, Italy; fabrizio.olivieri@unina.it (F.O.); salvatore.graci@unina.it (S.G.); silvana.francesca@unina.it (S.F.); mrigano@unina.it (M.M.R.)

**Keywords:** selection index, SolCAP genomic platform, reduced representation sequencing (RRS), high temperatures, resistance genes

## Abstract

The constitution of heat tolerant F_1_ hybrids is a challenge to ensure high yield and good fruit quality in the global climate. In the present work, we evaluated 15 genotypes for yield-related traits highly affected by high temperatures (HT). This phenotypic analysis allowed to identify four parental genotypes showing promising yield performances under HT conditions. Two of these genotypes also exhibited good fruit quality traits. A molecular marker analysis was carried out for six resistance genes to pathogens mostly affecting tomatoes. This analysis evidenced the presence of a maximum of three resistant alleles in parental genotypes. Exploring single nucleotide polymorphisms (SNPs) revealed by two high-throughput genotyping platforms allowed identifying additional 12 genes potentially involved in resistance to biotic stress, to be further investigated. Following these considerations, 13 F_1_ hybrids were constituted combining the parental genotypes and then evaluated for multiple traits under HT conditions. By estimating a hybrid index based on yield performances, desirable quality and resistance gene, we identified seven hybrids showing the best performances. The promising results obtained in the present work should be confirmed by evaluating the best hybrids selected for additional years and environments before proposing them as novel commercial hybrids that could maintain high performances under HT conditions.

## 1. Introduction

High temperatures (HT) and the predicted resulting global warming will affect several aspects of the ecosystems. As a whole, agriculture represents one of the human activities highly affected from these changes. In fact, the predicted increase of 2–3 °C in the seasonal temperature average [1] will require the adoption of several strategies to mitigate the negative effect of the harsh temperatures on crops. In particular, HT negatively affect plants during their entire life cycle, especially during reproductive phases, leading to harvest losses in several crops [2]. Tomato (*S. lycopersicum* L.) is one of the most widely grown vegetables worldwide and is regarded as a relevant functional food, widely used in the Mediterranean diet [3,4]. Notwithstanding its subtropical origin, tomato is negatively affected by HT. Indeed, temperatures over 35 °C during the reproductive phases, from pollen development to fruit set [5], lead to a dramatic drop of the final yield. In this context, research in tomato is today focused on the exploration of novel genetic resources tolerant to HT by carrying out phenotypic evaluations, which can represent a robust instrument for the selection of genotypes useful for breeding strategies. In particular, the recovery of old varieties as a source of genetic variability can help to develop F_1_ hybrids, carrying traits that may guarantee the improvement of final yields under HT conditions.

On the other hand, the increase of temperatures also leads to the onset of plant diseases [6], due to the improved growth conditions of pests under HT. Indeed, one of the issues in tomato cultivation is the proliferation of the whiteflies *Bemisia tabaci* that is reported to be more effective under increased temperatures [7]. This pest acts as vector for different virus diseases, such as the tomato yellow leaf curl virus (TYLCV), which is found to be highly dangerous in tropical and subtropical environments [8]. Moreover, climate change has a strong impact on the soil ecosystem, leading to the growth of dangerous parasites, such as nematodes, which are more aggressive at higher temperatures [9].

The use of F_1_ hybrids represents a powerful tool to combine in the same genotype favorable alleles at different loci, such as those controlling tolerance to biotic and abiotic stresses [10,11,12]. Nowadays, this goal can be successfully achieved by the aid of genotypic characterization of tomato parental lines of F_1_ hybrids by using different genotyping platforms belonging to the array-based and next-generation sequencing (NGS) technologies. The availability for tomato of the Solanaceae Coordinated Agricultural Project (SolCAP) SNP array allows the identification of effective SNPs for breeding applications [13]. Several works reported the use of this platform in genome-wide association studies (GWAS) for the identification of candidate genes involved in the tolerance to HT and for agronomical traits [14,15]. Moreover, the NGS technology known as reduced representation sequencing (RRS) permits one to investigate the genetic variability, building datasets of thousand SNP markers and reducing genotyping costs [16]. Among the RSS technologies, the genotyping-by-sequencing (GBS), an enzyme-based technology that reduces the genome representation, has the advantage to compare the discovered polymorphisms within the population of interest, extracting rapidly genotypic information [16]. Analogously, the double-digest restriction-site associated DNA sequencing (ddRAD-seq) technology uses two different restriction enzymes to construct the genomic library, generating more information on genetic variability and maximizing cost-effectiveness [17].

The aim of the present study is to develop F_1_ hybrids showing tolerance to HT combined with the capacity of contrasting plant diseases and maintaining good quality traits, using as parental lines some genotypes previously characterized for heat tolerance and quality traits [15,18,19]. Their genetic variability was explored and allowed the identification of candidate genes potentially involved in biotic stress responses. Cleaved amplified polymorphic sequences (CAPS) and sequence characterized amplified region (SCAR) markers targeting known resistance genes were also designed. This multilevel analysis allowed to develop different hybrid combinations. In the future, the promising F_1_ hybrids selected in this study will be evaluated in additional environments and years to select the most stable combinations to get commercial hybrids tolerant to HT.

## 2. Results and Discussion

A major challenge in agriculture in the global warming era is how to develop novel strategies to mitigate heat stress by maintaining high crop yield under elevated temperatures and contrasting the correlated onset of plant biotic diseases. In this context, new and traditional breeding tools could support programs aimed at pyramiding desirable traits in F_1_ hybrids, in terms of yield performances, quality traits and pathogens resistance. Therefore, the present work proposes a breeding approach that combines phenotypic and molecular analyses to obtain novel F_1_ hybrids exhibiting good performances and high fruit quality under HT. This work has been carried out using 14 tomato lines available in our laboratory and previously characterized for tolerance to HT and for fruit quality [15,19,20]. Among them, two genotypes (E103 and E111) did not show good yield under HT but good fruit quality when grown under standard environmental conditions. An additional line (PDLUC) was added as parental line in the crossing schemes for its very high good fruit quality traits [21]. These 15 lines were intercrossed in different combinations and tested again under HT together with the F_1_ hybrids obtained.

In order to characterize these parental lines, we exploited the use of different molecular markers. Using high-throughput genotyping platforms, the genetic variability exhibited by these lines was investigated at the whole genome level as well as at targeted resistance genes to different pathogens. Finally, using loci-specific markers we also explored the presence of resistant/susceptible alleles in six genes. All together, these analyses allowed us to in-depth characterize the parental lines and the F_1_ hybrids.

### 2.1. Phenotypic Evaluation of Parental Lines

**Figure 1:**
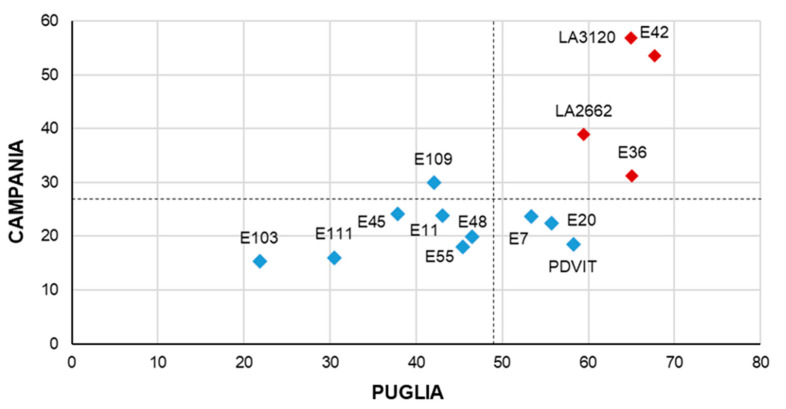
Scatter plot of the selection indexes (SI) calculated for 14 genotypes in the two experimental fields in Puglia (*X*-axis) and in Campania (*Y*-axis) pooling data recorded in the years 2016 and 2017.

Fourteen parental genotypes were previously grown and characterized under HT for two years (2016 and 2017) in two different locations (Campania and Puglia) in Southern Italy. Data concerning heat tolerance in terms of reproductive (FS) and yield (TNF, FW and YP) traits, reported in the years 2016 [15], 2017 [18] and in the present work (Table S1), were here pooled and used to estimate a selection index (SI) [15], calculated based on reproductive (FS) and yield traits evaluated in each experimental area (Figure 1). The SI varied between 22 (E103) and 68 (E42) in Puglia and between 15 (E103) and 57 (LA3120) in Campania. This analysis allowed the identification of four genotypes (E36, E42, LA2662 and LA3120) showing good yield performances in both the environments. In particular E36 and E42 confirmed the high SI previously reported [15] whereas the genotypes E103 and E111, which were selected for their good fruit quality [20] and not for heat tolerance, had the worst SI in both Campania and Puglia with values lower than the averages. 

Then, in the year 2019 all 15 parental genotypes were grown under HT, transplanting plants with one-month delay compared to the standard agronomic practices of the location, similarly to previous studies [15,18], thus imposing higher temperatures during flowering and fruit set stages, since it is reported that temperatures exceeding the 32 °C are critical during all the preharvest phases [1]. During the 2019, 72% of the growing season showed maximum temperatures higher than 32 °C and in the 33% of days the temperature even exceeded 35 °C. Indeed, only a window on nine days showed temperatures lower than 30 °C, assessing the high temperatures during the reproductive stages (Figure S1). 

Data regarding one reproductive trait (FS) and three yield-related traits (TNF, FW, and YP) evidenced that most genotypes maintained good FS percentages under HT, and that four genotypes (E7, E20, E36, and E48) confirmed YP values higher than 3.0 kg/plant (Table S2). Yield performances under HT cannot be the only final goal to get novel tolerant genotypes for the tomato market. Indeed, this trait should be combined with other desirable traits for the consumers and farmers. On one hand, climate change and raising temperatures can alter the fruit quality in tomato fruits, whereas the guarantee of good tasting and nutritional properties are also highly desirable [22]. On the other hand, besides the good performances under high temperatures, the availability in parental lines of variability in resistance genes potentially facing biotic stresses is also crucial to get novel F_1_ hybrids tolerant/resistant to pathogens [23]. For these reasons, in the present work the parental genotypes were also evaluated in the year 2019 for quality traits, measuring total soluble solid content (TSSC), titratable acidity (TA) and the TA/TSSC ratio. Most of the genotypes (12 out 15, 80.0%) exhibited a high or medium quality level when considering both marketable (TSSC) and nutritional traits (TA and TSSC/TA) (Table S2), usually confirming the good quality data already reported [20]. Indeed, in various studies the values of 5 for TSSC, 0.4 for TA and 12 for TSSC/TA ratio were considered as minimum thresholds for a good-tasting tomato [24,25]. 

### 2.2. Molecular Screening for Resistance Genes in Parental Lines

Since the HT also can enhance the proliferation of important vectors and negatively affect the immunity response of tomato plants [26], causing the increase of plant diseases due to ubiquitous pathogens or vector-mediated infections [27], the raising use of chemicals in agriculture would be required, thus favoring environmental pollution and the onset of pest resistances [28].

In terms of response to pathogen attack, the development of molecular markers targeting known resistance genes can help the selection of resistant genotypes [29]. Therefore, the screening of six well-known resistance genes was carried out on parental genotypes by using CAPS and SCAR markers (Table S3). Three of these markers (for the root-knot disease, Mi-1.2, tomato yellow leaf curl virus, Ty-3 and late blight disease, Ph-3) were already reported in the literature [30,31,32]. From the molecular screening, we found that all the parental genotypes showed the Mi-1.2 homozygous resistant allele, and in the global warming era, this would be very important due to the increased proliferation of nematodes under HT conditions [9]. As for the Ph-3 and Ty-3 genes, we found eight (E11, E36, E45, E48, E55, E109, E111 and PDLUC) and three (E55, PDLUC and PDVIT) genotypes evidencing the homozygous resistance alleles for Ph-3 and Ty-3, respectively. The first gene is usually incorporated in breeding programs [33], whereas the interest in the second one is crucial since the vector of the TYLCV, the white tobacco fly *Bemisia tabaci*, shows an increased proliferation under HT [7]. Three additional markers targeting the genes Sw-5, Tm-2 and Ve-1 were designed in the present work, deriving from the multiple alignment of resistant and susceptible alleles retrieved from GenBank accessions (Figures S2 and S3). As for the CAPS marker targeting the Sw-5 gene, the alignment among four different sequences permitted the identification of a missense substitution of a C (resistant variant) with a T (susceptible variant) at the position 68,085,710 (Tomato Genome version SL4.0, available at the Solgenomics Network, www.solgenomics.net; accessed on 2 September 2021). Two primers targeting a region that includes this mutation were designed and the expected amplicons of 358 bp were digested with the *Hpy188I* restriction enzyme. The enzymatic digestion pattern consisted of two fragments of 133 and 225 bp for the susceptible allele whereas three fragments of 43, 133 and 182 bp were produced for the resistant variant. Among the genotypes evaluated, no one showed the resistant allele. As for the Tm-2 gene, two SCAR markers, targeting alternatively the susceptible (Tm-2S) and resistant (Tm-2R) alleles were designed based on the identification of highly polymorphic regions by aligning sequences deriving from resistant and susceptible genotypes. The amplicon size of both Tm-2 markers was 206 bp and the genotypes showing expected amplicons in the two PCR reactions resulted in heterozygous condition. From the combined analysis on parental genotypes, we demonstrated that the genotypes E103 and PDVIT carried the Tm-2 resistant allele.

As for the Ve-1 gene, initially we performed a PCR using dCAPS primers designed on the mutation in position 60,264 bp, which discriminates the resistant (C variant) from the susceptible genotypes (G variant), following the digestion with the restriction enzyme *DdeI*. Unfortunately, the electrophoretic analysis revealed that the designed marker discriminated the homozygous resistant genotypes but did not distinguish the susceptible from the heterozygous ones. This probably resulted from the high identity percentage (91%), covering the 99% of the sequences, between the Ve-1 gene (*Solyc09g005090*) and the Ve-2 gene (*Solyc09g005080*), which probably would lead to the amplification of regions from both the genes. Therefore, since the two sequences are very similar, we studied the regions with lower identity percentages to design highly specific primers the Ve-1 gene in order to set up a nested PCR (Figure S3) to amplify the desired target only from the Ve-1 gene. Then, using the nested-PCR amplicon as template, we performed the second PCR using the dCAPS primer pair located in the internal region of the first template sequence. The expected amplicon size of the Ve-1 gene obtained by the first-PCR was 1016 bp, whereas the second one generated a fragment of 261 bp. For the dCAPS marker on the forward primer a A has been changed in a C at position 22, to permit the allele discrimination through a restriction enzyme reaction with *DdeI* (sequence recognized and cut: C/TNAG). Following the digestion, two fragments of 156 bp and 105 bp for the resistant allele or three fragments of 130, 105 and 26 bp for the susceptible allele were produced. Using this marker, we detected three genotypes (E11, E42 and E109) showing the resistant allele. 

### 2.3. Genomic Characterization of Parental Lines

A whole genome characterization of the parental genotypes was carried out using two high-throughput genotyping datasets, one deriving from a ddRAD experiment and the other from a SolCAP analysis, previously carried out in our laboratory [15,18,20]. Comprehensively, 37,554 and 7720 SNPs were investigated, respectively. This analysis allowed to reach two different objectives. The first was the quantification of the genetic distance among the genotypes, by estimating the Identity-by-State (IBS) score on each dataset (Table S4). In most comparisons the IBS values were higher than 90% in both the datasets, whereas they were lower when including E11, E55, and E109 (0.63–0.85), and decreased when including E42 or PDVIT (0.54–0.66). The lowest value of IBS (0.54) was observed when E42 and PDVIT were compared, thus indicating that these two genotypes were the most genetically distant [34]. The second objective was the investigation of the presence in these genotypes of additional genes potentially involved in the resistance to pathogens. This investigation was performed by exploring the genetic variability of genes reported in the PRGdb (http://www.prgdb.org/prgdb; accessed on 2 September 2021), which is an accessible open-source bioinformatic platform holding more than 16,000 known and putative R-genes belonging to 192 plant species [35]. Out of 1516 genes retrieved from the database for tomato, 761 were polymorphic in our datasets respect to the reference genome (SL4.0 cv Heinz1706).

Among the genotypes, E11 was the most polymorphic with 38 out of the 71 SNPs detected and 53.5% polymorphic genes, followed by E42 and PDVIT with more than 40% polymorphic loci and E55 with approx. 38%; on the contrary LA2662 showed no SNPs. The prediction of the SNP effect was derived for these polymorphic loci, in order to identify mutations in genes involved in the resistance to biotic stress. We identified SNPs with moderate and high impact on the protein function in most parental genotypes, which could consequently suggest the tolerance/resistance of these genotypes to different pathogens, besides those evidenced by the molecular marker analysis described above. In particular, four SNPs (0.53%) showed high impact on the protein function. As for the other SNPs, 91 (12.0%) and 595 (78.2%) showed low and modifier impacts on proteins, respectively, whereas 71 (9.3%) had a moderate effect (Table S5).

Regarding the 71 SNPs mapping on coding regions of 57 resistance genes (Table 1), three genes were polymorphic in most of the genotypes. Among them, *Solyc02g093100*, coding for a leucine-rich protein, was polymorphic in eight genotypes whereas *Solyc04g008650* and *Solyc07g055670* coding for a leucine-rich receptor and for a lectin domain receptor, respectively, in six genotypes. Moreover, SNPs showing predicted high impact on protein function were found in four genes. In particular, three of them code for protein kinases (*Solyc04g082510*, *Solyc06g060690* and *Solyc06g068920*) and the last one codes for a receptor-like serine-threonine-protein kinase (*Solyc07g062040*). Regarding other genes carrying moderate impact variants in some of the parental genotypes, *Solyc01g087200*, coding for a generic disease resistance protein, is reported to be the target of a micro-RNA, which modulates the immunity against *Phytophtora infestans* [36]. Other two genes coding for two kinases (*Solyc04g057930* and *Solyc05g053930*) were identified in RNA-seq experiment as downregulated genes in a Sw-7 resistant line [37]. On chromosome 11, the gene *Solyc11g011180*, coding for a LRR receptor-like serine/threonine-protein kinase, was reported to be responsible for the resistance to *Fusarium oxysporium f.s. lycopersici* [38]. Finally, the gene *Solyc09g011320*, coding for a Serine/threonine-protein kinase, was found to be part of a gene cluster putatively involved in the tolerance to *Fusarium oxysporium f.s. radicis lycopersici* [39]. The role of these potential resistance genes in contrasting pathogens causing diseases will be further investigated in the parental genotypes, by applying resistance tests or molecular marker analysis.

All phenotypic and genotypic data obtained in this study on the parental genotypes are reported in Table 2: out of 15 selected genotypes, three (E36, LA2662 and LA3120) showed high heat tolerance both in terms of fruit set and yield production, five (E45, E103, E111, PDLUC and PDVIT) showed desirable qualitative traits, other two (E42 and PDVIT) showed high source of whole-genome genetic variability, three (E11, E42, PDVIT) exhibited high or medium variability in potential resistance genes, and five genotypes (E11, E55, E109, PDLUC, PDVIT) carry the maximum of three resistance genes. Therefore, the interactions of these genotypes in different cross combinations could lead to the production of F_1_ hybrids exhibiting good performances for many desirable traits.

### 2.4. Multilevel Evaluation of F_1_ Hybrids

**Figure 2:**
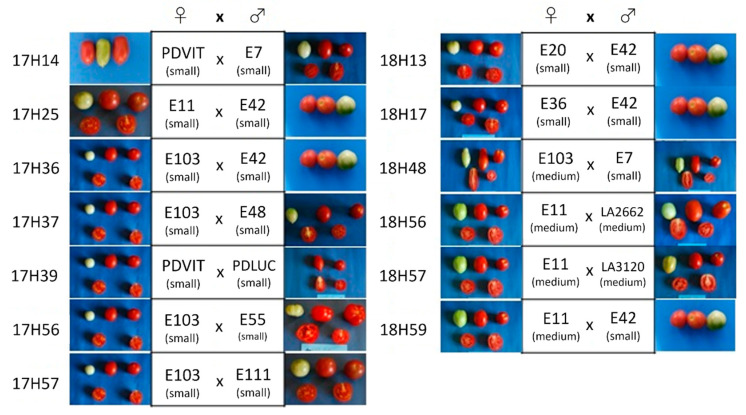
Thirteen F_1_ hybrid combinations obtained using 15 different parental genotypes.

The 13 F_1_ hybrids obtained from different cross combinations involving the fifteen parental genotypes (Figure 2) were also evaluated in the year 2019. Data concerning the parental lines have been already reported in a previous paragraph, whereas we here compare those recorded for the hybrids (Table S6).

**Figure 3:**
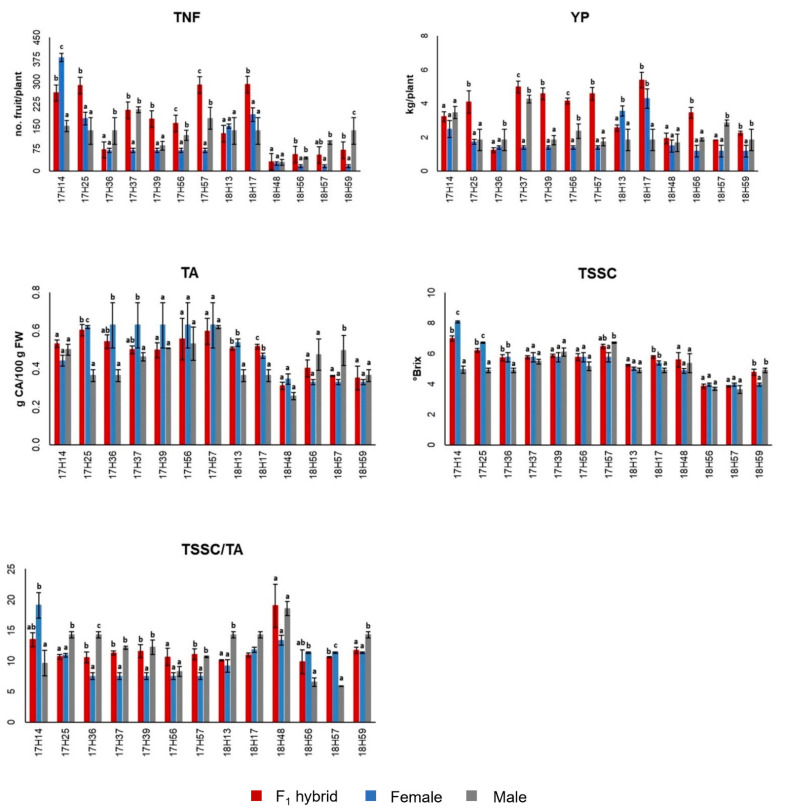
Comparisons among the F_1_ hybrids and their parents by Duncan’s t-test (a, b, c, groups of significance). TNF, number of fruit per plant, YP yield per plant, TSSC, total soluble solid content, TA, titratable acidity and TSSC/TA ratio, FW, fruit weight.

As for the hybrids, they usually exhibited FS values higher than 50%, and six of them performed more than 4.0 kg/plant, mostly due to a very high production of fruit/plant. In order to compare each F_1_ hybrid with its parents, the ANOVA and post-hoc Duncan’s tests were applied to each combination for the productive and qualitative traits. The analysis evidenced variable results for each trait (Figure 3; Table S7). 

As for TNF, five hybrids (17H25, 17H39, 17H56, 17H57 and 18H17) showed a significant increase compared to the parental genotypes and eight (17H14, 17H36, 17H37, 18H13, 18H48, 18H56, 18H57 and 18H59) were statistically similar to at least one parent. For FW, only three combinations (17H37, 17H39 and 17H56) showed a significant increase whereas in most cases (17H14, 17H57, 18H13, 18H17, 18H56 and 18H59) the FW resulted intermediate between the two parents. As for YP, six hybrids (17H25, 17H37, 17H39, 17H56, 17H57 and 18H56) had values statistically higher than both parental genotypes, showing a significant 3-, 4-fold increase. These results underlined a substantial improvement of the yield components in the hybrids respect to the parents [40,41,42]. Although the yield, as well as the yield-related traits, are important aspects for selecting genotypes tolerant under HT, these must be combined with other good qualitative traits desirable for consumers [40]. As for the TSSC, in most cases the F_1_ hybrids showed values that were intermediate between the two parents (17H14, 17H25, 17H36, 17H37, 17H39, 17H57, 18H56, 18H57 and 18H59), whereas only three hybrids (18H13, 18H17 and 18H48) showed values higher than both parental genotypes, although not statistically significant. This behavior is in agreement with findings from Solieman et al. [40], who observed similar trends for this trait. For TA, most of the hybrids did not show a statistical increase or decrease while only the hybrid 18H17 showed a value significantly higher than both parents. Finally, for the derived-trait TSSC/TA ratio, similarly to previous qualitative traits, most of the hybrids showed values that ranged between those of their parents and only three (17H56, 17H57 and 18H48) showed values higher than both parents. This trait is generally an indicator of the palatability and flavor, and, in all cases, we observed values similar or higher than 10, corresponding to the best ratio reported in literature [25].

Due to the significant increase detected in some traits for some F_1_ hybrid combination, the mid-parent heterosis was calculated to quantify the magnitude of the increases (Table 3), and it indicated a global positive heterotic effect for many traits. 

As for the TNF, the Het% varied from −30.4% (17H36) to 136.3% (17H57), whereas for FW the Het% ranged from −31.3% (18H57) and 27.1% (17H39). As for YP, the values recorded varied from −24.2% (17H36) to a maximum of 192.7% (17H57), with other four hybrids 17H25 (127.8%), 17H39 (183.1%), 17H56 (120.8%) and 18H56 (126.2%) exceeding the 100% of heterosis. In addition, the two hybrids 17H37 and 18H17 exhibited values of heterosis higher than 75%. These results suggested that heterosis is greater in TNF and YP compared with FW, and that the higher values of YP even under HT depends on TNF and not on FW, confirming data reported by other authors when grown tomato under standard environmental conditions [43,44]. In particular, Hannan et al. [45] found very high heterosis for yield (189%) in a hybrid, a value comparable with the 193% we observed in the hybrid 17H57. These results reflect the trend of the mid-parents heterosis recorded in the F_1_ hybrids and could depend on the effect of the interaction of numerous additive genes [41], which favors the increase of the yield-related traits, even under HT.

Regarding the fruit quality traits, the Het% recorded in TSSC varied from −1.12% (17H39) to 12.5% (18H17), with most hybrids showing a moderate heterotic effect. As for TA, the Het% varied from −20.8% (18H57) to 32.2% (18H17), with most hybrids showing a negative heterotic effect. Finally, considering the index TSSC/TA, the Het% ranged from −16.0% (18H17) and 35.2% (17H56), with hybrids 17H56, 17H57 and 18H57 showing good values.

Finally, the presence of resistant alleles for four genes (Ph-3, Tm-2, Ty-3, and Ve-1) was verified in all the F_1_ hybrids using the designed molecular markers. As expected, eight hybrids (17H25, 17H37, 17H56, 17H57, 18H17, 18H56, 18H57 and 18H59) carried Ph-3 resistant allele at the heterozygous condition and one (18H48) in homozygous condition, whereas six hybrids (17H14, 17H36, 17H37, 17H39, 17H56 and 17H57) carried the resistance gene variant of Tm-2 in heterozygous condition. Finally, for Ty-3 and Ve-1 we found three (17H14, 17H39 and 17H56) and seven hybrids (17H25, 17H36, 18H13, 18H17, 18H48, 18H56 and 18H57) in heterozygous condition for the resistant allele. Moreover, 18H59 showed the Ve-1 resistant allele in homozygous condition. The Mi-1.2 and Sw-5 markers were excluded from the genotypic analysis of the F_1_ hybrids due to the homozygous condition of their parents for these loci. Regarding the Mi-1.2 all the F_1_ hybrids had the resistant allele in homozygous condition whereas for the Sw-5 no parents and, consequently, F_1_ hybrids showed the resistant allele. The use of molecular markers designed for targeting resistance genes also allowed to verify the hybrid nature of the progeny of each cross.

**Figure 4:**
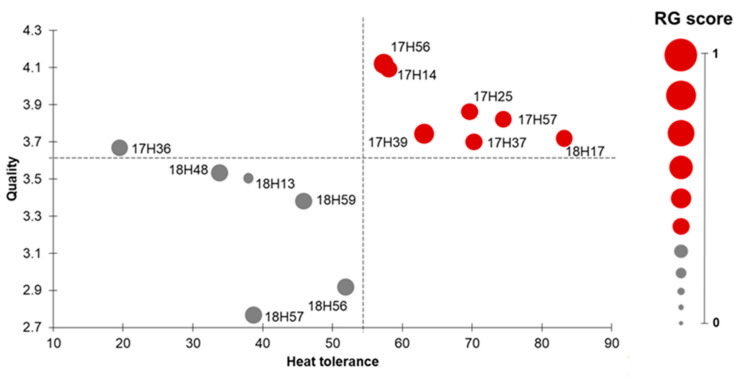
Scatter plot of hybrid scores considering the quality, heat tolerance and resistance genes of the 13 F_1_ hybrids by conferring an arbitrary score for each phenotypic trait reported. Dashed lines represented the mean threshold for each trait. The red spots represented the acceptable Scale for the RG score.

As a whole, we summarize the results observed in the F_1_ hybrids for all the traits, both in terms of response to high temperature and of resistance to biotic stresses and fruit quality (Figure 4), estimating a hybrid index (HI). This takes into account three different scores: the heat tolerance score (HTS), the quality score (QS) and the resistance genes score (RGS). Indeed, among the hybrids, seven showed scores higher than the mean threshold for each analysed trait. Among them, 17H39 and 17H56 showed the highest score for the resistance genes detected by molecular markers (RGS = 0.7) whereas the hybrid 18H17 showed the highest score for the heat tolerance trait (HTS = 83.0). Finally, eight genotypes showed quality score (QS) higher than the threshold of 3.6 and the hybrids 17H14 and 17H56 showed the highest score (QS = 4.1). Since the pyramiding breeding approach has the final aim of cumulating in a single genetic background many desirable traits for farmers and consumers, we summarized our results in a scatter plot, evidencing the three most important parameters for the present study, that are heat tolerance, quality traits and resistance genes. The scatter plot divided the hybrids in two macro-groups. This analysis revealed that seven F_1_ hybrids (17H14, 17H25, 17H37, 17H39, 17H56, 17H57 and 18H17) showed a good compromise between yield components, qualitative traits and resistance genes. Surprisingly, some of these heat tolerant F_1_ hybrids derived from crosses involving two parental genotypes, previously selected only for their quality traits, such as E103 × PDLUC (17H39) and E103 × E111 (17H57). These results should be confirmed by evaluating the best hybrids for additional years and environments, before proposing them as novel commercial hybrids that could maintain high performances under adverse environmental conditions. 

## 3. Materials and Methods

### 3.1. Plant Material 

Fifteen parental genotypes were grown in the year 2019 in an experimental field in Acerra (Campania, 40°96′52″ N, 14°42′86″ E) together with 13 F_1_ hybrids deriving from different cross combinations, obtained in the year 2018. The list of parental genotypes is reported in Table S8. All plants were grown in a randomized complete block design consisting of three replicates and ten plants per replicate and transplanted in open field with one-month delay respect to the standard agronomic practices, in order to increase the possibility to expose the plants to high temperatures during the reproductive phases.

### 3.2. Phenotypic Evaluation

During the growing season, four yield-related and three fruit quality traits were evaluated: fruit set (FS), number of fruit per plant (TNF), fruit weight (FW), yield per plant (YP), total soluble solid content (TSSC) and titratable acidity (TA) and then, the TSSC/TA ratio. Ten fruits at red ripe stage from each replicate and for each genotype were harvested and stored to −80 °C until qualitative analysis. The TSSC was measured using a refractometer (HANNA Instruments, Smithfield, RI, USA), whereas the TA was measured by using a Fiveasy F20 digital pH meter (Mettler Toledo, Columbus, OH, USA). Three fruits from each replicate and for each genotype were disrupted and centrifugated for 20 min at 6000 rpm to collect the surnatant. Thirty milliliters of double-distilled water were added to 2.5 g of surnatant and put on a stirrer at the speed of 100 rpm. Then, 25 mL of NaOH (0.1 N) solution was charged on an analytical burette and dropped in the solution until the breakpoint of pH = 8.0. The formula TA (g CA/100 g) = [NaOH] (N) × vol_NaOH_ (mL)/m_sample_ (g) × 1000/10 = G1 was used for the quantification of titratable acidity, where CA corresponds to the citric acid monohydrate, [NaOH] corresponds to the concentration of titratant expressed in Normality, vol_NaOH_ corresponds to the added volume to the solution of titration, m_sample_ the quantity in grams of the surnatant used in the solution of titration. Finally, to calculate the titratable acidity, the G1 was multiplied for 0.070, corresponding to the fixed factor for the determination of CA content.

### 3.3. Statistical Analysis

All the statistical analyses were carried out using IBM SPSS v. 23 (IBM Corp., Armonk, NY, USA). In order to evaluate the 14 genotypes, a selection index was calculated for each genotype based on the sum of the different score of the arbitrary scale assigned to the three traits FS, TNF, YP: for FS and TNF 0 = trait value ranging from 0 to 10, 1 = from 11 to 20, 2 = from 21 to 30, 3 = from 31 to 40 etc.; for YP, the scale was applied by multiplying the kg/plant value by 10, as reported in Ruggieri et al. [15]. Then, in order to compare F_1_ hybrids and their parents, ANOVA analysis and post-hoc Duncan test were carried out to evidence the statistical significance among them. In order to estimate the heterotic effect of each trait in the F_1_ hybrids, the heterosis percentage (%Het) was calculated using the formula Het% = (F_1t_ − PM_t_)/PM_t_, where F_1t_ is the value recorded for the hybrid for trait t and PM_t_ is the mean of the two parents for trait t. In order to select elite hybrids, a hybrid index (HI) was estimated, considering three different scores: the heat tolerance score (HTS) calculated as HTS = (TNF/10 + YP ∗ 10) [15]; the quality score (QS) calculated by dividing each evaluated trait for the optimum values retrieved in bibliography [24,46] using the formula QS = TA/0.3 + TSSC/5.5 + TSSC/(TA ∗ 12.5); the resistance score (RGS) where RGS = n. of resistant allele/n. of assayed molecular markers. For each score, a mean threshold corresponding to the average score of the hybrids was calculated in order to select the elite hybrids. The scores and their mean thresholds obtained were represented in a scatter plot.

### 3.4. Genomic Analysis

In order to carry out the genotyping analysis of all the parental lines, two different sequencing datasets were used: one retrieved from previous studies [15,20] based on the platform 7.7K SolCAP single nucleotide polymorphism (SNP) array and the other deriving from the ddRAD sequencing technology, here applied. In this last case, DNA extraction and quantification, sample preparation and sequencing were carried out according to Olivieri et al. [18].

The demultiplexing step of raw reads was performed using Stacks v2.0 [47]. The alignment to the reference genome of *Solanum lycopersicum* cv Heinz (Tomato Genome version SL4.0, available at the Solgenomics Network, www.solgenomics.net; accessed on 2 September 2021) was carried out using BWA-MEM (Burrow-Wheeler Alignment—Maximal Exact Matches) through the software Samtools 1.6 [48] with default parameters, selecting reads that map to a single location. The detection of loci from the aligned reads, the SNP calling and the generation of variant calling format (.vcf) file were carried out using the software Bcftools 1.6 (http://samtools.github.io/bcftools/bcftools.html; accessed on 2 September 2021) [49]. The filtering step was performed using VcfTools v.0.1.13 (http://vcftools.sourceforge.net; accessed on 2 September 2021) [50] by setting the maximum missing value (max-missing) = 0.5 and minimum mean of Depth of Coverage (min-mean DP) = 5. All non-polymorphic loci were manually eliminated. Then, in order to quantify the genetic distance among the genotypes, pairwise comparisons were carried out by calculating the Identity-by-State (IBS) allele-sharing indexes using the software PLINK v.1.90b5.2 [51,52], represented by a correlation matrix describing the genetic distance for each comparison. Finally, resistance genes were identified by querying the PRG database (available at http://www.prgdb.org/prgdb; accessed on 2 September 2021) [35] and searched for in the genotyping datasets. The polymorphic alleles were those showing alternative allele to the reference tomato genome (SL4.0 cv Heinz 1706).

### 3.5. Marker Design and Analysis

In order to transfer in the F_1_ hybrids resistance genes to common biotic diseases in tomato, a marker-assisted screening was carried out on the parental genotypes. At this purpose, six resistance genes were investigated: root-knot nematode resistance (Mi-1.2) [30], late blight resistance (Ph-3) [30], tomato yellow leaf curl virus (Ty-3) [31], tomato spotted wilt disease (Sw-5), tomato mosaic virus (Tm-2) and *Verticillium* wilt disease resistance 1 (Ve-1) (Table S9). For genes Mi-1.2, Ph-3, and Ty-3, markers were derived from the literature, whereas for the other three genes molecular markers were designed and reported in the present work. In particular, the CAPS for the Sw-5 gene was designed based on a SNP mutation within the genomic region of *Solyc09g098130*, identified following a multiple alignment between four accessions of susceptible and resistant tomato genotypes (Genbank accessions: AY007366.1, FJ686039.1, FJ686040.1, FJ686042.1). As for the Tm-2, two different primer pairs were designed to distinguish the susceptible variant (using the primer pair coded as Tm-2S_SCAR) from the resistant one (coded as Tm-2R_SCAR). The SCAR markers targeting the Tm-2 were designed in a high polymorphic region of *Solyc09g018220*, aligning resistant and susceptible genotypes (Genbank accessions AF536199.1 and AF536200.1, respectively). Finally, Ve-1 gene was investigated designing a dCAPS marker. A multiple alignment between eleven accessions of susceptible (Genbank accessions FJ464555.1, FJ809925.1, FJ809926.1 and FJ686045.1) and resistant tomato genotypes (Genbank accessions AF272367.1, NM_001247545.2, FJ809928.1, FJ809927.1, FJ464557.1, FJ464556.1 and FJ464553.1) was carried out to detect the susceptible and resistant variants. As a whole, markers used are CAPS, dCAPS and SCAR, and are listed in Table S9. The PCR cycle was set up as follow: 95 °C for 5 min, 35 cycles at 95 °C for 30 s, annealing temperature depending on the primer pair for 30 s and 72 °C for 30 s, a final extension of 7 min at 72 °C. For the evaluation of PCR amplicons, a 1.2% standard agarose-gel electrophoresis analysis was performed to assess the amplification at expected size. Following the restriction enzyme digestion, the agarose-gel concentration varied from 1.5 to 3.0% depending on the resolution required to discriminate the fragments.

## 4. Conclusions

We evaluated 13 F_1_ hybrids under HT conditions in order to select those combining good yield performances with quality traits and resistance to pathogens, by designing a novel strategy of phenotypic and genotypic characterization of the parental lines involved. A selection index based on phenotypic responses for yield-related traits under HT conditions was used to characterize the 14 parental lines. A molecular markers analysis targeting six specific resistance genes facing the most common disease evidenced the possibility of pyramiding up to four resistant alleles in the same hybrid. In addition, a whole genome characterization of these lines allowed us to identify polymorphisms in 57 genes potentially involved in the response to various pathogens, and this result will be exploitable in the future to increase the number of resistance genes in F_1_ hybrids actually under production in our laboratory. Following this multi-trait evaluation of the parental lines, the 13 hybrids were evaluated under HT, and we used a hybrid index based on yield performances, tolerance to pathogens and fruit quality traits to select the best ones. With this view in mind, we identified seven hybrids (17H14, 17H25, 17H37, 17H39, 17H56, 17H57 and 18H17) showing higher hybrid indexes and high heterotic effect, especially for yield-related traits. The promising results obtained in the present work represent an initial proof of the application of the methodology proposed and could be complemented in further studies.

## Figures and Tables

**Table 1:** List of 71 SNPs with high (in bold) and moderate impact detected in 57 resistance genes reported in the PRG database (prgdb.org; accessed on 2 September 2021). The locus and the protein function were reported together with the genotypes carrying each mutated gene respect to the reference genome Heinz1706 (www.solgenomics.net; accessed on 2 September 2021). When more than one SNP was detected in the same gene for some genotypes, the number of SNPs is reported in brackets.

Gene	Mutated Genotype (no. SNPs)	Predicted Effect	Protein Function
*Solyc01g014520*	E42	missense_variant	Receptor-like protein kinase
*Solyc01g074010*	E11, E42	missense_variant	Protein kinase domain
*Solyc01g080880*	E20, E42, PDVIT	missense_variant	Protein kinase domain
*Solyc01g087200*	E42, E55	missense_variant	Disease resistance protein
*Solyc02g070000*	E11, E36, E42, E55	missense_variant	Leucine-rich receptor-like protein kinase family protein
*Solyc02g078780*	PDVIT	missense_variant	Protein STRUBBELIG-RECEPTOR FAMILY 3
*Solyc02g093100*	E7, E11, E36, E42, E45, E48, E55, PDVIT	missense_variant	Leucine-rich repeat protein kinase family protein
*Solyc04g007030*	E11, E36, E42	missense_variant	Disease resistance protein
*Solyc04g008650*	E7. E11, E20, E36, E42, E45	missense_variant	Inactive leucine-rich repeat receptor-like serinethreonine-protein kinase
*Solyc04g014400*	E11, E36, E42, E55	missense_variant	LRR receptor-like serine/threonine-protein kinase GSO1
*Solyc04g015130*	E42, E55, PDVIT	missense_variant	Protein kinase G11A
*Solyc04g049400*	E42, E55, PDVIT	missense_variant	Protein kinase domain
*Solyc04g054200*	E42, E55, PDVIT	missense_variant	Leucine-rich receptor-like protein kinase family protein
*Solyc04g057930*	E11 (2), E42 (2), E55 (2) E109, PDVIT (2)	missense_variant	Pkinase domain-containing protein/Usp domain-containing protein
*Solyc04g074270*	E11 (3), PDVIT (3)	missense_variant	Outer arm dynein light chain 1
***Solyc04g082510***	**E55** (2)	missense_variant; **stop_gained**	**Protein kinase**
*Solyc04g082620*	E55 (2)	missense_variant	Serine/threonine-protein kinase/endoribonuclease IRE1a
*Solyc05g009740*	E7, E11 (2), E20, E45, E109 (2)	missense_variant	Disease resistance protein
*Solyc05g009800*	E11, E109	missense_variant	Leucine-rich repeat receptor-like serine/threonine-protein kinase
*Solyc05g013280*	LA3120 (2)	missense_variant	Pseudomonas resistance
*Solyc05g050700*	E11, E36, E42, E109, PDVIT	missense_variant	Leucine-rich repeat protein
*Solyc05g051050*	E11, E36, E42, E109	missense_variant	Shaggy-related protein kinase theta
*Solyc05g053010*	E11, E109, PDVIT	missense_variant	Lectin receptor kinase
*Solyc05g053930*	E55	missense_variant	Protein kinase APK1B, chloroplastic
*Solyc05g054340*	E55, PDVIT	missense_variant	Plant resistance protein
*Solyc06g036470*	E55	missense_variant	G-type lectin S-receptor-like serine/threonine-protein kinase
***Solyc06g060690***	**E11**, **E55**, **PDVIT**	**stop_gained**	**Protein kinase superfamily protein**
***Solyc06g068920***	**E7**, **E20**, **E36**, **E42**, **E48**	**stop_lost**	**Protein kinase domain**
*Solyc06g072340*	E11, E36, E45, E55, E109	missense_variant	Protein kinase domain
*Solyc07g007140*	E11, E42, E48, E55, E109	missense_variant	MAP kinase kinase kinase 44
*Solyc07g053010*	E42, PDVIT	missense_variant	Disease resistance protein
*Solyc07g053300*	E42	missense_variant	ABC transporter G family member 10
*Solyc07g053910*	E55	missense_variant	Protein kinase domain
*Solyc07g055670*	E11, E42, E45, E48, E55, PDVIT	missense_variant	Lectin-domain receptor-like kinase
***Solyc07g062040***	**E42**, **E55**	**stop_gained**	**Receptor-like serine/threonine-protein kinase**
*Solyc08g081210*	E20, E36, E42, PDVIT	missense_variant	MAP kinase kinase kinase 66
*Solyc09g005080*	E36, PDVIT	missense_variant	Verticillium resistance
*Solyc09g007110*	E36 (5), E45 (5), E48 (5), PDVIT (5)	missense_variant	Leucine-rich receptor-like protein kinase family protein
*Solyc09g011320*	E11, E109	missense_variant	Serine/threonine-protein kinase
*Solyc09g074240*	E11, E36, E109, PDVIT	missense_variant	Protein kinase domain
*Solyc09g091580*	E11, E55	missense_variant	Protein kinase domain
*Solyc09g091990*	E42, E48, PDVIT	missense_variant	Receptor like protein kinase S.2
*Solyc10g005140*	E55	missense_variant	probably inactive receptor-like protein kinase At2g46850
*Solyc10g084390*	E55	missense_variant	Protein kinase superfamily protein
*Solyc11g007280*	E42	missense_variant	Pleiotropic drug resistance protein 2
*Solyc11g011080*	E11 (3), E36 (3), E109 (3)	missense_variant	Disease resistance protein (TIR-NBS-LRR class)
*Solyc11g011090*	E11 (2), E36, E109 (2)	missense_variant	Disease resistance protein (TIR-NBS-LRR class)
*Solyc11g011180*	E11, E36, E109	missense_variant	Lrr receptor-like serinethreonine-protein kinase gso1
*Solyc11g013880*	E11	missense_variant	G-type lectin S-receptor-like serine/threonine-protein kinase
*Solyc11g018690*	E11, E42, PDVIT	missense_variant	ABC transporter G family member 25
*Solyc11g020230*	E11, PDVIT	missense_variant	Serine/threonine-protein kinase-like protein CCR4
*Solyc11g020280*	E11, PDVIT	missense_variant	Receptor-like protein kinase
*Solyc11g033270*	E11, PDVIT	missense_variant	MAP kinase kinase kinase 82
*Solyc11g042990*	E11, PDVIT	missense_variant	Kinase protein
*Solyc11g056680*	E11, PDVIT	missense_variant	Leucine-rich repeat receptor-like protein
*Solyc12g016220*	E55	missense_variant	Disease resistance protein
*Solyc12g021280*	E11, E36, E45, E48, E55	missense_variant	Serine/threonine-protein kinase STN7, chloroplastic

**Table 2:** Summary of the phenotypic analyses and the molecular screening of resistance genes carried out in 15 parental genotypes. The resistant allele detection is reported for six genes: Mi-1.2, Root-knot nematode resistance; Ph-3, late blight resistance; Sw-5, Tomato spotted wilt virus; Tm-2, Tomato mosaic virus resistance; Ty-3, Tomato yellow leaf curl virus resistance; Ve-1, Verticillium wilt resistance. Scores of heat tolerance, fruit quality traits, genetic distance, variability in PRG database of parental genotypes are also reported. HH, very-high; H, high; M, medium; L, low.

Genotype	Heat Tolerance		Fruit Quality ^3^	Genetic Distance ^4^	Variability In Prg Database ^5^	Resistance Allele
	FS ^1^	YP ^2^				
E7	HH	M	M	L	L	Mi-1.2
E11	M	M	M	M	H	Mi-1.2, Ph-3, Ve-1
E20	M	M	M	L	L	Mi-1.2
E36	H	H	M	L	M	Mi-1.2, Ph-3
E42	M	M	M	H	H	Mi-1.2, Ve-1
E45	M	M	H	L	L	Mi-1.2, Ph-3
E48	M	M	M	L	L	Mi-1.2, Ph-3
E55	H	M	L	M	M	Mi-1.2, Ph-3, Ty-3
E103	HH	L	H	-	-	Mi-1.2, Tm-2
E109	M	M	M	M	L	Mi-1.2, Ph-3, Ve-1
E111	M	L	H	-	-	Mi-1.2, Ph-3
LA2662	H	H	L	L	-	Mi-1.2
LA3120	H	H	L	L	L	Mi-1.2
PDLUC	-	-	H	-	-	Mi-1.2, Ph-3, Ty-3
PDVIT	H	M	H	H	H	Mi-1.2, Tm-2, Ty-3

^1^ For fruit set: HH ≥ 70%; H = 50–70%; M = 30–50%; ^2^ For yield production: H ≥ 3.0 kg/pt; M = 2.0–3.0 kg/pt; L ≤ 2.0 kg/pt; ^3^ For fruit quality: H = both market and nutritional good quality traits; M = marketable or nutritional good quality traits; L = neither marketable nor nutritional good quality traits [22]; ^4^ For genetic distance: H = average IBS < 0.7; M = 0.7 < average IBS < 0.9; L = average IBS > 0.9; ^5^ For genetic variability in PRG database: H ≥ 40% of polymorphic PRGs; M = 20–40%; L ≤ 20%.

**Table 3:** Heterosis percentage (Het%) of six phenotypic traits in 13 F_1_ hybrids. TNF, number of fruit per plant, FW, fruit weight, YP, yield per plant, TSSC total soluble solid content, TA titratable acidity, TSSC/TA total soluble solid content/titratable acidity ratio. The green and brown colours and their shades represent the lower and the higherpositive and negative heterotic levels, respectively.

Hybrid	Trait					
	TNF	FW	YP	TSSC	TA	TSSC/TA
17H14	−1.37	−21.44	8.00	7.16	14.41	−6.02
17H25	85.10	9.34	127.76	7.03	20.13	−15.17
17H36	−30.35	−10.65	−24.21	7.50	−0.98	−2.93
17H37	49.28	15.08	76.14	2.22	−12.74	15.17
17H39	128.27	27.05	183.09	−1.12	−16.92	16.92
17H56	69.83	22.42	120.76	5.49	−17.80	35.21
17H57	136.32	18.49	192.65	3.63	−12.12	20.04
18H13	−11.71	8.12	−5.52	5.88	16.54	−13.52
18H17	79.27	4.99	75.77	12.48	32.23	−16.01
18H48	9.60	12.70	22.04	9.45	2.72	18.94
18H56	80.34	13.33	126.21	0.87	−6.18	9.95
18H57	−4.79	−31.29	−8.24	1.32	−20.79	22.71
18H59	−6.97	−24.50	47.79	7.52	20.64	−8.63

## Data Availability

The data presented in this study are available on request from the corresponding author.

## Supplementary Materials

The following are available online at https://www.mdpi.com/article/10.3390/plants10102168/s1, Figure S1. Temperature variation observed in Acerra during the summer 2019. Daily maximum (red), daily mean (blue) temperatures. Dashed red line represents the critical threshold of 32 °C. Figure S2. Multiple alignment of two resistance genes for the discrimination of resistant and susceptible variants. (A) *Tomato spotted wilt virus* resistance—Sw-5; (B) *Tomato mosaic virus* resistance—Tm-2. The blue boxes represent the coding regions of the genes, the orange boxes represent the UTR regions. Polymorphisms are evidenced by red rings. Figure S3. Design of the nested and dCAPS markers targeting the Ve-1 gene. A) ClustalW alignment between the Ve-1 gene resistant allele (FJ464557.1), Ve-1 susceptible allele (FJ464555.1) and Ve-2 gene (AF365929.1). The red and blue arrows correspond to the primer pairs for the nested PCR and dCAPS marker, respectively. The red box evidenced the SNP associated with susceptible/resistant phenotype. The position of the mismatch introduced in the forward primer is also reported (in red bold). B) Electrophoretic analysis of the dCAPS marker designed for Ve-1 gene on 4 hybrids and their parental lines. Table S1. Phenotypic data of selected parental genotypes evaluated in 2017. FS, fruit set; TNF, no. fruit per plant; FW, fruit weight; YP, yield per plant, are reported. Table S2. Phenotypic data of 15 parental genotypes evaluated in 2019. TNF, no. fruit per plant; FW, fruit weight; YP, yield per plant; TSSC, total soluble solid content; TA, titratable acidity; CA, citric acid. Table S3. Summary of molecular screening of resistance genes in 15 parental genotypes. The resistant (R, in red) and susceptible (S, in yellow) alleles are reported for six genes: Mi-1.2, *Root-knot nematode* resistance; Ph-3, late blight resistance; Sw-5, *Tomato spotted wilt virus*; Tm-2, *Tomato mosaic virus* resistance; Ty-3, *Tomato yellow leaf curl virus* resistance; Ve-1, *Verticillium wilt* resistance. Table S4. Identity-by-state values describing the pairwise comparisons to estimate the genetic distance among the genotypes. Lower values indicated higher genetic distances. The white color indicates missing values in the analysis. Table S5. Overview of the number of SNPs mapping on genes involved in the response to resistance diseases. The number of SNPs were grouped based on the categorical classification of the impact of the mutation. Table S6. Phenotypic data of 13 F_1_ hybrids evaluated in 2019. FS, fruit set; TNF, no. fruit per plant; FW, fruit weight; YP, yield per plant; TSSC, total soluble solid content; TA, titratable acidity; CA, citric acid. Table S7. One-way ANOVA performed for each trait and for each source of variation. FS, fruit set, TNF, no. fruit per plant; FW, fruit weight; YP, yield per plant; TSSC, total soluble solid content; TA, titratable acidity, d.f., degrees of freedom. Table S8. Parental genotypes used in the crossing schemes for the constitution of F_1_ hybrids. Experiment trials: C16, Campania 2016, C17, Campania 2017, C19, Campania 2019, P16, Puglia 2016, P17, Puglia 2017. Selected trait: HT, Heat-tolerance, Q, Quality. Table S9. List of molecular markers designed for each resistance gene. The primer sequences, restriction enzymes and references are also reported.

## References

[1] Sato S., Peet M.M., Gardner R.G. (2004). Altered flower retention and developmental patterns in nine tomato cultivars under elevated temperature. Sci. Hortic..

[2] Hasanuzzaman M., Nahar K., Fujita M., Ahmad P., Chandna R., Prasad M., Ozturk M. (2013). Enhancing plant productivity under salt stress: Relevance of poly-omics. Salt Stress Plants.

[3] Aliberti A., Olivieri F., Graci S., Rigano M.M., Barone A., Ruggieri V. (2020). Genomic Dissection of a Wild Region in a Superior Solanum pennellii Introgression Sub-Line with High Ascorbic Acid Accumulation in Tomato Fruit. Genes.

[4] Farinetti A., Zurlo V., Manenti A., Coppi F., Mattioli A.V. (2017). Mediterranean diet and colorectal cancer: A systematic review. Nutrition.

[5] Paupière M.J., van Haperen P., Rieu I., Visser R.G., Tikunov Y.M., Bovy A.G. (2017). Screening for pollen tolerance to high temperatures in tomato. Euphytica.

[6] Bailey-Serres J., Parker J.E., Ainsworth E.A., Oldroyd G.E., Schroeder J.I. (2019). Genetic strategies for improving crop yields. Nature.

[7] Ramos R.S., Kumar L., Shabani F., Picanço M.C. (2019). Risk of spread of tomato yellow leaf curl virus (TYLCV) in tomato crops under various climate change scenarios. Agric. Syst..

[8] Lapidot M., Polston J.E. (2006). Resistance to Tomato yellow leaf curl virus in tomato. Natural Resistance Mechanisms of Plants to Viruses.

[9] Trudgill D., Tzortzakakis E. (2005). A comparative study of the thermal time requirements for embryogenesis in Meloidogyne javanica and M. incognita. Nematology.

[10] Panthee D.R. (2020). ‘Mountain Crown’: Late Blight and Tomato mosaic virus-resistant Plum Hybrid Tomato and Its Parent, NC 1 Plum. HortScience.

[11] Prabhandakavi P., Pogiri R., Kumar R., Acharya S., Esakky R., Chakraborty M., Pinnamaneni R., Palicherla S.R. (2020). Pyramiding Ty-1/Ty-3, Ty-2, ty-5 and ty-6 genes into tomato hybrid to develop resistance against tomato leaf curl viruses and recurrent parent genome recovery by ddRAD sequencing method. J. Plant Biochem. Biotechnol..

[12] Vijeth S., Dhaliwal M.S., Jindal S.K., Sharma A. (2018). Evaluation of tomato hybrids for resistance to leaf curl virus disease and for high-yield production. Hortic.Environ. Biotechnol..

[13] Hamilton J.P., Sim S.C., Stoffel K., Van Deynze A., Buell C.R., Francis D.M. (2012). Single nucleotide polymorphism discovery in cultivated tomato via sequencing by synthesis. Plant Genome.

[14] Bauchet G., Grenier S., Samson N., Segura V., Kende A., Beekwilder J., Cankar K., Gallois J.L., Gricourt J., Bonnet J. (2017). Identification of major loci and genomic regions controlling acid and volatile content in tomato fruit: Implications for flavor improvement. New Phytol..

[15] Ruggieri V., Calafiore R., Schettini C., Rigano M.M., Olivieri F., Frusciante L., Barone A. (2019). Exploiting genetic and genomic resources to enhance heat-tolerance in tomatoes. Agronomy.

[16] Poland J.A., Rife T.W. (2012). Genotyping-by-sequencing for plant breeding and genetics. Plant Genome.

[17] Peterson B.K., Weber J.N., Kay E.H., Fisher H.S., Hoekstra H.E. (2012). Double digest RADseq: An inexpensive method for de novo SNP discovery and genotyping in model and non-model species. PLoS ONE.

[18] Olivieri F., Calafiore R., Francesca S., Schettini C., Chiaiese P., Rigano M.M., Barone A. (2020). High-Throughput Genotyping of Resilient Tomato Landraces to Detect Candidate Genes Involved in the Response to High Temperatures. Genes.

[19] Sacco A., Ruggieri V., Parisi M., Festa G., Rigano M.M., Picarella M.E., Mazzucato A., Barone A. (2015). Exploring a tomato landraces collection for fruit-related traits by the aid of a high-throughput genomic platform. PLoS ONE.

[20] Ruggieri V., Francese G., Sacco A., D’Alessandro A., Rigano M.M., Parisi M., Milone M., Cardi T., Mennella G., Barone A. (2014). An association mapping approach to identify favourable alleles for tomato fruit quality breeding. BMC Plant Biol..

[21] Tranchida-Lombardo V., Aiese Cigliano R., Anzar I., Landi S., Palombieri S., Colantuono C., Bostan H., Termolino P., Aversano R., Batelli G. (2018). Whole-genome re-sequencing of two Italian tomato landraces reveals sequence variations in genes associated with stress tolerance, fruit quality and long shelf-life traits. DNA Res..

[22] Scarano A., Olivieri F., Gerardi C., Liso M., Chiesa M., Chieppa M., Frusciante L., Barone A., Santino A., Rigano M.M. (2020). Selection of tomato landraces with high fruit yield and nutritional quality under elevated temperatures. J. Sci. Food Agric..

[23] Gimenez E., Salinas M., Manzano-Agugliaro F. (2018). Worldwide research on plant defense against biotic stresses as improvement for sustainable agriculture. Sustainability.

[24] Beckles D.M. (2012). Factors affecting the postharvest soluble solids and sugar content of tomato (*Solanum lycopersicum* L.) fruit. Postharvest Biol. Technol..

[25] Kader A., Lyons J., Morris L. (1974). Postharvest responses of vegetables to preharvest field temperature. HortScience.

[26] Janda T., Khalil R., Tajti J., Pál M., Darkó É. (2019). Responses of young wheat plants to moderate heat stress. Acta Physiol. Plant..

[27] Gorovits R., Moshe A., Ghanim M., Czosnek H. (2013). Recruitment of the host plant heat shock protein 70 by Tomato yellow leaf curl virus coat protein is required for virus infection. PLoS ONE.

[28] Schuster D.J., Mann R.S., Toapanta M., Cordero R., Thompson S., Cyman S., Shurtleff A., Morris R.F. (2010). Monitoring neonicotinoid resistance in biotype B of Bemisia tabaci in Florida. Pest Manag. Sci. Former. Pestic. Sci..

[29] Foolad M.R., Panthee D.R. (2012). Marker-assisted selection in tomato breeding. Crit. Rev. Plant Sci..

[30] Jung J., Kim H.J., Lee J.M., Oh C.S., Lee H.-J., Yeam I. (2015). Gene-based molecular marker system for multiple disease resistances in tomato against Tomato yellow leaf curl virus, late blight, and verticillium wilt. Euphytica.

[31] Kim M., Park Y., Lee J., Sim S.-C. (2020). Development of molecular markers for Ty-2 and Ty-3 selection in tomato breeding. Sci. Hortic..

[32] Santos D., da Silva P.M., Abrantes I., Maleita C. (2020). Tomato Mi-1.2 gene confers resistance to Meloidogyne luci and M. ethiopica. Eur. J. Plant Pathol..

[33] Ren Z., You Z., Munir S., Zhang Y., Li H., Zhang J., Wang T., Zheng W., Ye Z. (2019). Development of a highly specific co-dominant marker for genotyping the Ph-3 (tomato late blight resistance) locus by comparing cultivated and wild ancestor species. Mol. Breed..

[34] Vela-Avitúa S., Meuwissen T.H., Luan T., Ødegård J. (2015). Accuracy of genomic selection for a sib-evaluated trait using identity-by-state and identity-by-descent relationships. Genet. Sel. Evol..

[35] Cruz C.M., Paytuvi-Gallart A., Di Donato A., Sundesha V., Andolfo G., Aiese Cigliano R., Sanseverino W., Ercolano M.R. (2017). PRGdb 3.0: A comprehensive platform for prediction and analysis of plant disease resistance genes. Nucleic Acids Res..

[36] Jiang N., Cui J., Hou X., Yang G., Xiao Y., Han L., Meng J., Luan Y. (2020). Sl-lncRNA15492 interacts with Sl-miR482a and affects *Solanum lycopersicum* immunity against *Phytophthora infestans*. Plant J..

[37] Chellappan P., Qiyue M., Reza S., Stewart K.S., Hutton S.F., Scott J.W., Zhangjun F., Kai-Shu L. (2019). Comprehensive transcriptome analysis and functional characterization of PR-5 for its involvement in tomato Sw-7 resistance to tomato spotted wilt tospovirus. Sci. Rep..

[38] Catanzariti A.M., Do H.T., Bru P., de Sain M., Thatcher L.F., Rep M., Jones D.A. (2017). The tomato I gene for Fusarium wilt resistance encodes an atypical leucine-rich repeat receptor-like protein whose function is nevertheless dependent on SOBIR 1 and SERK 3/BAK 1. Plant J..

[39] Devran Z., Kahveci E., Hong Y., Studholme D.J., Tör M. (2018). Identifying molecular markers suitable for Frl selection in tomato breeding. Theor. Appl. Genet..

[40] Solieman T., El-Gabry M., Abido A. (2013). Heterosis, potence ratio and correlation of some important characters in tomato (*Solanum lycopersicum* L.). Sci. Hortic..

[41] Tamta S., Singh J. (2018). Heterosis in tomato for growth and yield traits. Int. J. Veg. Sci..

[42] Yadav S.K., Singh B., Baranwal D., Solankey S. (2013). Genetic study of heterosis for yield and quality components in tomato (*Solanum lycopersicum*). Afr. J. Agric. Res..

[43] Avdikos I.D., Tagiakas R., Tsouvaltzis P., Mylonas I., Xynias I.N., Mavromatis A.G. (2021). Comparative Evaluation of Tomato Hybrids and Inbred Lines for Fruit Quality Traits. Agronomy.

[44] Kumari S., Sharma M.K. (2011). Exploitation of heterosis for yield and its contributing traits in tomato, *Solanum lycopersicum* L.. Int. J. Farm Sci..

[45] Hannan M., Ahmed M., Razvy M., Karim R., Khatun M., Haydar A., Hossain M., Roy U. (2007). Heterosis and correlation of yield and yield components in tomato (*Lycopersicon esulentum* Mill.). Am.-Eurasian J. Sci. Res..

[46] George B., Kaur C., Khurdiya D., Kapoor H. (2004). Antioxidants in tomato (*Lycopersium esculentum*) as a function of genotype. Food Chem..

[47] Catchen J., Hohenlohe P.A., Bassham S., Amores A., Cresko W.A. (2013). Stacks: An analysis tool set for population genomics. Mol. Ecol..

[48] Li H., Durbin R. (2009). Fast and accurate short read alignment with Burrows–Wheeler transform. Bioinformatics.

[49] Li H. (2011). A statistical framework for SNP calling, mutation discovery, association mapping and population genetical parameter estimation from sequencing data. Bioinformatics.

[50] Danecek P., Auton A., Abecasis G., Albers C.A., Banks E., DePristo M.A., Handsaker R.E., Lunter G., Marth G.T., Sherry S.T. (2011). The variant call format and VCFtools. Bioinformatics.

[51] Chang C.C., Chow C.C., Tellier L.C., Vattikuti S., Purcell S.M., Lee J.J. (2015). Second-generation PLINK: Rising to the challenge of larger and richer datasets. Gigascience.

[52] Purcell S., Neale B., Todd-Brown K., Thomas L., Ferreira M.A., Bender D., Maller J., Sklar P., De Bakker P.I., Daly M.J. (2007). PLINK: A tool set for whole-genome association and population-based linkage analyses. Am. J. Hum. Genet..

