# Supplementary material for: Accelerating the Development of Heat Tolerant Tomato Hybrids through a Multi-Traits Evaluation of Parental Lines Combining Phenotypic and Genotypic Analysis
Source: Plants (Basel). 2021 Oct 13;10(10):2168. doi: 10.3390/plants10102168 (PMC8539001; doi:10.3390/plants10102168)
Supplement: Supplementary file 1 [file plants-10-02168-s001.zip › Figure S1.pptx]

## Slide 1
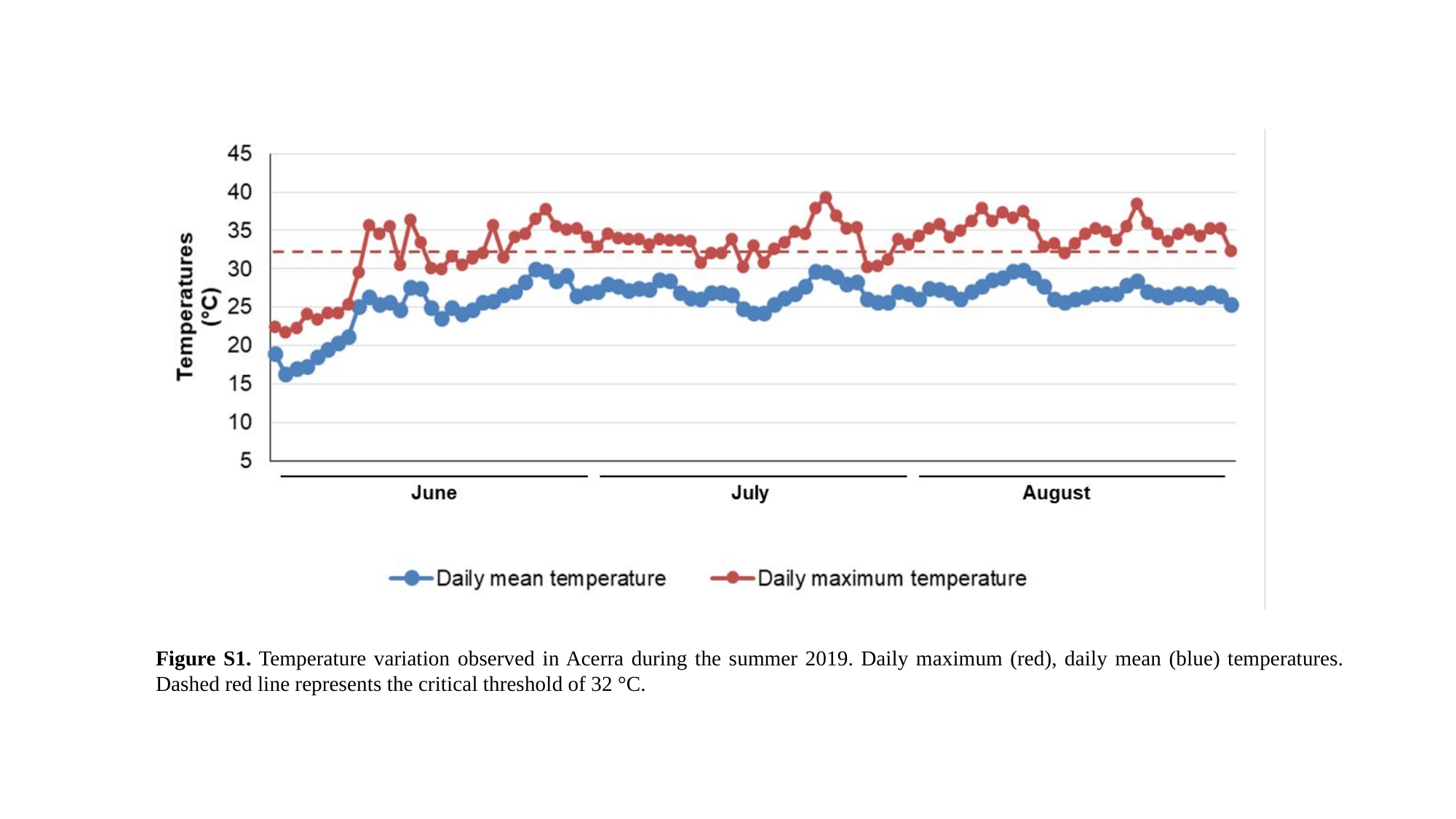

Figure S1. Temperature variation observed in Acerra during the summer 2019. Daily maximum (red), daily mean (blue) temperatures. Dashed red line represents the critical threshold of 32 °C.
