# Supplementary material for: Accelerating the Development of Heat Tolerant Tomato Hybrids through a Multi-Traits Evaluation of Parental Lines Combining Phenotypic and Genotypic Analysis
Source: Plants (Basel). 2021 Oct 13;10(10):2168. doi: 10.3390/plants10102168 (PMC8539001; doi:10.3390/plants10102168)
Supplement: Supplementary file 1 [file plants-10-02168-s001.zip › Figure S2.pptx]

## Slide 1
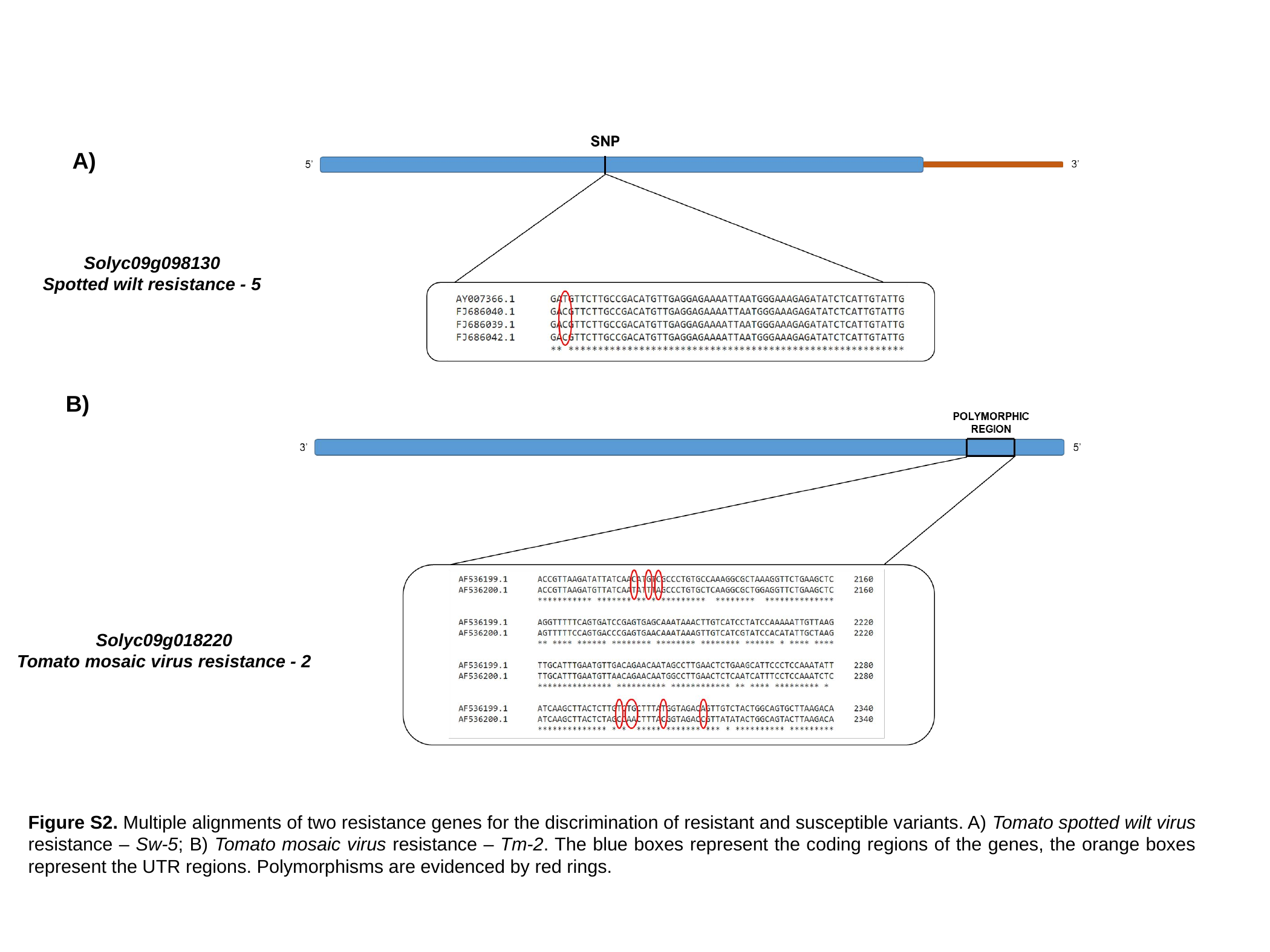

A)
Solyc09g098130
Spotted wilt resistance - 5
B)
Solyc09g018220
Tomato mosaic virus resistance - 2
Figure S2. Multiple alignments of two resistance genes for the discrimination of resistant and susceptible variants. A) Tomato spotted wilt virus resistance – Sw-5; B) Tomato mosaic virus resistance – Tm-2. The blue boxes represent the coding regions of the genes, the orange boxes represent the UTR regions. Polymorphisms are evidenced by red rings.
