# Supplementary material for: Accelerating the Development of Heat Tolerant Tomato Hybrids through a Multi-Traits Evaluation of Parental Lines Combining Phenotypic and Genotypic Analysis
Source: Plants (Basel). 2021 Oct 13;10(10):2168. doi: 10.3390/plants10102168 (PMC8539001; doi:10.3390/plants10102168)
Supplement: Supplementary file 1 [file plants-10-02168-s001.zip › Figure S3.pptx]

## Slide 1
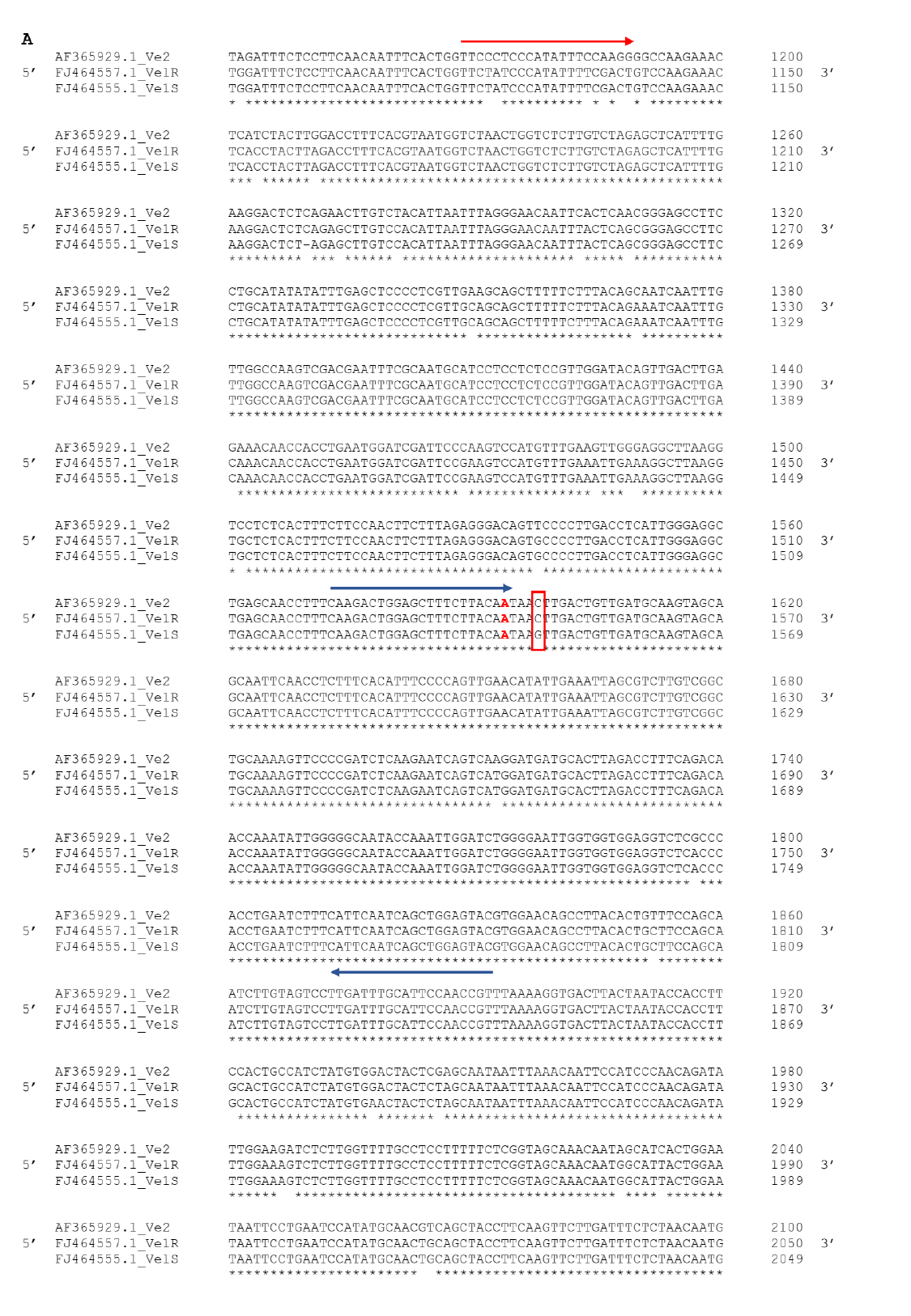

## Slide 2
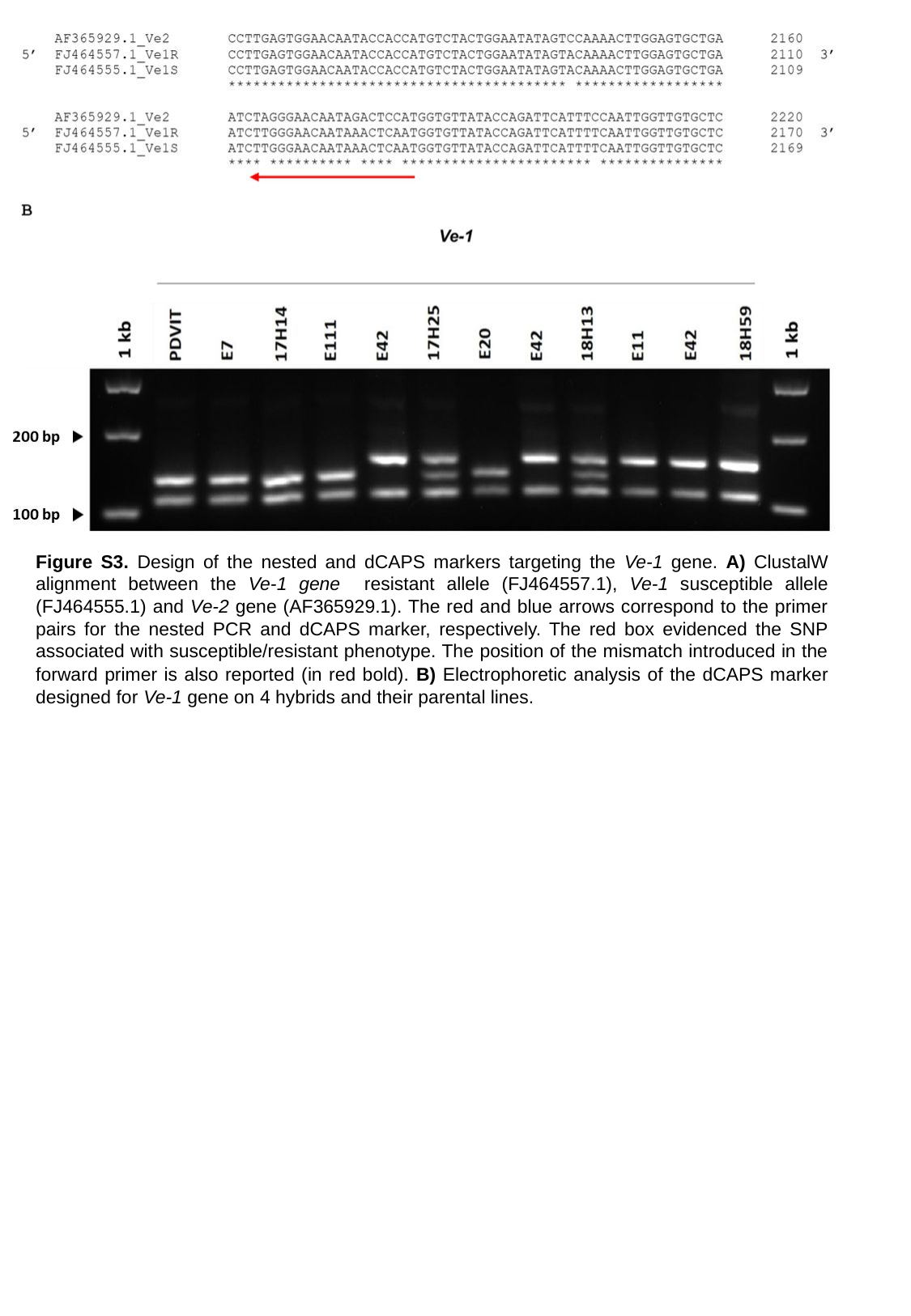

Figure S3. Design of the nested and dCAPS markers targeting the Ve-1 gene. A) ClustalW alignment between the Ve-1 gene resistant allele (FJ464557.1), Ve-1 susceptible allele (FJ464555.1) and Ve-2 gene (AF365929.1). The red and blue arrows correspond to the primer pairs for the nested PCR and dCAPS marker, respectively. The red box evidenced the SNP associated with susceptible/resistant phenotype. The position of the mismatch introduced in the forward primer is also reported (in red bold). B) Electrophoretic analysis of the dCAPS marker designed for Ve-1 gene on 4 hybrids and their parental lines.
