# Supplementary material for: Accelerating the Development of Heat Tolerant Tomato Hybrids through a Multi-Traits Evaluation of Parental Lines Combining Phenotypic and Genotypic Analysis
Source: Plants (Basel). 2021 Oct 13;10(10):2168. doi: 10.3390/plants10102168 (PMC8539001; doi:10.3390/plants10102168)
Supplement: Supplementary file 1 [file plants-10-02168-s001.zip › Table S1.pdf]

Table S1. Phenotypic data of selected parental genotypes evaluated in 2017. FS, fruit set; TNF, no. fruit per plant; FW, fruit weight; YP, yield per plant are reported.

CAMPANIA 2017									
Genotype	FS (%)		TNF (no.)		FW (g)		YP (kg/pt)		Reference
	Mean	SE	Mean	SE	Mean	SE	Mean	SE	
E7	71.90	3.50	35.84	16.06	29.51	1.38	1.42	0.77	[6]
E11	34.72	0.14	37.13	0.89	77.37	10.12	2.86	0.32	
E20	25.89	7.44	57.60	9.50	29.35	3.83	2.24	0.46	
E36	69.65	3.99	87.31	10.93	26.07	0.84	2.52	0.12	[6]
E42	60.45	4.53	304.18	8.07	11.27	0.07	3.43	0.07	[6]
E45	56.35	5.84	62.27	11.92	40.95	13.69	2.79	0.44	[6]
E48	5.84	3.20	61.67	5.22	25.45	1.42	1.56	0.12	
E55	62.04	2.09	45.76	4.54	16.63	0.94	0.76	0.10	
E103	53.23	11.60	52.32	9.72	28.56	3.50	1.05	0.17	
E109	42.85	8.59	58.78	6.47	58.17	3.37	2.93	0.27	
E111	51.74	12.62	56.98	9.94	31.08	3.83	1.18	0.19	
LA2662	56.06	7.94	55.46	3.04	49.65	4.27	2.78	0.38	
LA3120	63.47	1.93	179.63	29.75	24.35	3.54	3.25	0.57	
PDVIT	47.55	2.09	94.50	1.44	9.90	1.75	0.43	0.19	
Mean	50.12		84.96		32.74		2.09		
Dev.st	18.00		72.62		18.49		0.99		

PUGLIA 2017									
Genotype	FS (%)		TNF (no.)		FW (g)		YP (kg/pt)		Reference
	Mean	SE	Mean	SE	Mean	SE	Mean	SE	
E7	61.21	5.94	161.53	13.04	24.68	2.68	3.99	0.57	[6]
E11	38.38	10.44	164.85	15.95	41.95	7.57	3.22	0.19	
E20	43.47	6.00	100.58	16.81	29.57	2.49	5.46	0.77	
E36	55.72	5.48	254.27	39.78	22.82	2.24	5.67	0.58	[6]
E42	25.84	0.29	417.25	47.32	10.20	0.67	4.22	0.39	[6]
E45	39.54	14.97	76.00	23.24	36.70	5.75	1.53	0.16	[6]
E48	42.21	7.36	170.60	4.42	21.61	0.97	3.69	0.24	
E55	49.13	5.35	181.43	19.57	19.51	2.65	3.44	0.09	
E103	53.46	16.04	70.15	11.23	29.63	3.24	1.44	0.20	
E109	30.91	0.52	64.06	20.52	60.11	26.07	5.40	0.46	
E111	42.73	15.85	85.19	13.26	37.12	4.76	1.89	0.27	
LA2662	54.22	2.58	112.17	19.99	49.29	3.62	4.28	0.47	
LA3120	43.50	5.78	229.18	12.48	25.84	3.81	3.77	0.64	
PDVIT	57.67	7.40	227.23	22.94	12.99	1.40	2.98	0.51	
Mean	45.57		165.32		30.14		3.64		
Dev.st	10.24		96.21		13.80		1.37		
