# Supplementary material for: Accelerating the Development of Heat Tolerant Tomato Hybrids through a Multi-Traits Evaluation of Parental Lines Combining Phenotypic and Genotypic Analysis
Source: Plants (Basel). 2021 Oct 13;10(10):2168. doi: 10.3390/plants10102168 (PMC8539001; doi:10.3390/plants10102168)
Supplement: Supplementary file 1 [file plants-10-02168-s001.zip › Table S2.pdf]

Table 2. Phenotypic data of 15 parental genotypes evaluated in 2019. TNF, no. fruit per plant; FW, fruit weight; YP, yield per plant; TSSC, total soluble solid content; TA, titratable acidity; CA, citric acid.

Genotype	TNF (no.)		FW (g)		YP (kg/pt)		TSSC (°Brix)		TA (g CA/100 g FW)		TSSC/TA	
	mean	SE	mean	SE	mean	SE	mean	SE	mean	SE	mean	SE
E7	151.09	10.93	23.94	0.24	3.47	0.21	4.95	0.24	0.50	0.21	9.99	0.94
E11	16.71	5.01	70.40	3.06	1.18	0.35	3.97	0.09	0.34	0.14	12.10	0.74
E20	150.83	7.54	24.38	0.22	3.55	0.31	5.02	0.09	0.55	0.22	9.37	0.47
E36	189.58	23.65	24.22	0.40	4.28	0.58	5.38	0.14	0.48	0.28	11.81	4.83
E42	134.97	25.67	13.67	0.37	1.85	0.37	4.90	0.15	0.33	0.14	13.55	0.73
E45	29.62	5.80	56.40	1.15	1.66	0.30	5.37	0.62	0.26	0.11	18.53	7.59
E48	205.75	5.77	21.89	0.29	4.26	0.13	5.48	0.15	0.45	0.18	11.85	0.30
E55	119.35	9.88	21.89	0.44	2.37	0.25	5.17	0.30	0.62	0.25	10.44	2.21
E103	68.79	7.37	21.68	0.31	1.40	0.10	5.77	0.32	0.74	0.31	10.13	2.64
E109	25.30	5.26	58.00	2.23	1.49	0.37	4.87	0.15	0.36	0.15	14.17	0.87
E111	176.71	21.27	10.84	0.27	1.73	0.14	6.72	0.04	0.63	0.36	10.92	4.46
LA2662	44.04	1.82	42.67	2.27	1.87	0.05	3.70	0.10	0.55	0.23	8.46	1.94
LA3120	95.95	3.46	29.73	1.76	2.84	0.10	3.63	0.26	0.57	0.23	7.94	2.00
PDLUC	84.94	8.44	21.53	0.54	1.83	0.16	6.10	0.28	0.51	0.21	12.00	0.55
PDVIT	381.49	13.84	8.87	0.21	2.49	0.51	8.08	0.07	0.43	0.18	18.39	1.09
