# Supplementary material for: Accelerating the Development of Heat Tolerant Tomato Hybrids through a Multi-Traits Evaluation of Parental Lines Combining Phenotypic and Genotypic Analysis
Source: Plants (Basel). 2021 Oct 13;10(10):2168. doi: 10.3390/plants10102168 (PMC8539001; doi:10.3390/plants10102168)
Supplement: Supplementary file 1 [file plants-10-02168-s001.zip › Table S3.pdf]

Table S3. Summary of molecular screening of resistance genes in 15 parental genotypes. The resistant (R, in red) and susceptible (S, in yellow) alleles are reported for six genes: *Mi-1.2*, *Root-knot nematode* resistance; *Ph-3*, late blight resistance; *Sw-5*, *Tomato spotted wilt virus*; *Tm-2, Tomato mosaic virus resistance*; *Ty-3*, *Tomato yellow leaf curl virus* resistance; *Ve-1*, *Verticillium wilt* resistance.

GENOTYPE	RESISTANCE GENES					
	Mi-1.2	*Ph-3*	*Sw-5*	*Tm-2*	*Ty-3*	*Ve-1*
E7	R	S	S	S	S	S
E11	R	R	S	S	S	R
E20	R	S	S	S	S	S
E36	R	R	S	S	S	S
E42	R	S	S	S	S	R
E45	R	R	S	S	S	S
E48	R	R	S	S	S	S
E55	R	R	S	S	R	S
E103	R	S	S	R	S	S
E109	R	R	S	S	S	R
E111	R	R	S	S	S	S
LA2662	R	S	S	S	S	S
LA3120	R	S	S	S	S	S
PDLUC	R	R	S	S	R	S
PDVIT	R	S	S	R	R	S
