# Supplementary material for: Accelerating the Development of Heat Tolerant Tomato Hybrids through a Multi-Traits Evaluation of Parental Lines Combining Phenotypic and Genotypic Analysis
Source: Plants (Basel). 2021 Oct 13;10(10):2168. doi: 10.3390/plants10102168 (PMC8539001; doi:10.3390/plants10102168)
Supplement: Supplementary file 1 [file plants-10-02168-s001.zip › Table S4.pdf]

Table S4. Identity-by-state values describing the pairwise comparisons to estimate the genetic distance among the genotypes. Lower values indicated higher genetic distances. The white color indicates missing values in the analysis.

	E7	E11	E20	E36	E42	E45	E48	E55	E109	PDVIT	LA2662	LA3120
**E7**	1											
**E11**	0.76	1										
**E20**	0.97	0.76	1									
**E36**	0.96	-	0.96	1								
**E42**	0.66	-	0.65	0.66	1							
**E45**	0.96	0.77	0.95	0.96	0.65	1						
**E48**	0.96	0.76	0.96	0.98	0.66	0.96	1					
**E55**	0.82	0.71	0.82	0.83	0.66	0.83	0.84	1				
**E109**	0.84	0.85	0.84	-	-	0.84	0.84	0.75	1			
**PDVIT**	0.61	-	0.61	0.61	0.54	0.61	0.61	0.63	-	1		
**LA2662**	0.94	-	0.95	0.95	0.66	0.96	0.95	0.82	-	0.61	1	
**LA3120**	0.94	-	0.96	0.96	0.66	0.95	0.96	0.83	-	0.61	0.95	1
