# Supplementary material for: Accelerating the Development of Heat Tolerant Tomato Hybrids through a Multi-Traits Evaluation of Parental Lines Combining Phenotypic and Genotypic Analysis
Source: Plants (Basel). 2021 Oct 13;10(10):2168. doi: 10.3390/plants10102168 (PMC8539001; doi:10.3390/plants10102168)
Supplement: Supplementary file 1 [file plants-10-02168-s001.zip › Table S6.pdf]

Table S6. Phenotypic data of 13 F_1 hybrids evaluated in 2019. FS, fruit set; TNF, no. fruit per plant; FW, fruit weight; YP, yield per plant; TSSC, total soluble solid content; TA, titratable acidity; CA, citric acid.

F_1 hybrid	TNF (no.)		FW (g)		YP (kg/pt)		TSSC (°Brix)		TA (g CA/100 g FW)		TSSC/TA	
	mean	SE	mean	SE	mean	SE	mean	SE	mean	SE	mean	SE
17H14	262.65	26.97	12.89	0.41	3.22	0.29	6.98	0.17	0.53	0.22	13.21	0.72
17H25	288.46	37.41	13.40	2.01	4.07	0.66	6.22	0.13	0.57	0.23	10.37	0.38
17H36	70.97	6.54	15.79	1.94	1.23	0.14	5.73	0.20	0.53	0.22	10.65	0.51
17H37	204.92	9.79	25.07	0.37	4.98	0.35	5.75	0.10	0.52	0.21	11.52	0.30
17H39	175.46	5.17	27.44	1.22	4.57	0.34	5.87	0.09	0.52	0.22	11.89	0.70
17H56	159.77	12.57	26.67	1.25	4.15	0.17	5.77	0.22	0.67	0.38	10.65	4.46
17H57	290.08	33.83	19.27	0.18	4.57	0.37	6.47	0.14	0.53	0.30	11.05	4.55
18H13	126.16	9.72	20.57	0.57	2.55	0.19	5.25	0.05	0.51	0.21	10.39	0.27
18H17	290.92	32.46	19.89	0.22	5.39	0.46	5.78	0.09	0.53	0.22	11.20	0.32
18H48	30.10	5.40	64.47	4.87	1.92	0.32	5.60	0.47	0.32	0.13	18.17	2.17
18H56	54.78	3.67	64.07	1.45	3.45	0.31	3.87	0.15	0.42	0.18	9.81	1.11
18H57	53.63	0.82	34.40	0.70	1.85	0.07	3.85	1.57	0.36	0.15	10.61	4.33
18H59	70.56	3.47	31.73	0.67	2.24	0.12	4.77	0.20	0.41	0.17	14.50	2.82
