# Supplementary material for: Accelerating the Development of Heat Tolerant Tomato Hybrids through a Multi-Traits Evaluation of Parental Lines Combining Phenotypic and Genotypic Analysis
Source: Plants (Basel). 2021 Oct 13;10(10):2168. doi: 10.3390/plants10102168 (PMC8539001; doi:10.3390/plants10102168)
Supplement: Supplementary file 1 [file plants-10-02168-s001.zip › Table S7.pdf]

Table S7. One-way ANOVA performed for each trait and for each source of variation. FS, fruit set, TNF, no. fruit per plant; FW, fruit weight; YP, yield per plant; TSSC, total soluble solid content; TA, titratable acidity, d.f., degrees of freedom.

Trait	Source of variation	Sum of Square	d.f.	Mean square	F	Sign.
FS	Genotypes	8142.28	17	478.96	2.84	0.00
	Parents	2253.80	8	281.73	1.63	0.19
	Hybrids	5264.11	8	658.01	4.01	0.01
	Between	624.37	1	624.37	2.39	0.13
	Error	6073.74	36	168.71		
	Total	14216.02	53			
TNF	Genotypes	674599.02	27	24985.15	30.68	0.00
	Parents	303391.58	14	21670.83	46.16	0.00
	Hybrids	322643.51	12	26886.96	21.52	0.00
	Between	48563.94	1	48563.94	5.67	0.02
	Error	42347.97	52	814.38		
	Total	716946.99	79			
FW	Genotypes	24601.41	27	911.16	132.72	0.00
	Parents	14183.96	14	1013.14	202.09	0.00
	Hybrids	10384.25	12	865.35	95.22	0.00
	Between	33.19	1	33.19	0.11	0.74
	Error	377.61	55	6.87		
	Total	24979.01	82			
YP	Genotypes	127.38	27	4.72	15.44	0.00
	Parents	44.72	14	3.19	11.80	0.00
	Hybrids	59.88	12	4.99	14.22	0.00
	Between	22.78	1	22.78	14.89	0.00
	Error	16.20	53	0.31		
	Total	143.58	80			
TA	Genotypes	0.62	27	0.02	8.31	0.00
	Parents	0.45	14	0.03	12.62	0.00
	Hybrids	0.17	12	0.01	4.75	0.01
	Between	0.00	1	0.00	0.00	0.96
	Error	0.07	24	0.00		
	Total	0.68	51			
Brix	Genotypes	84.09	27	3.11	20.90	0.00
	Parents	54.99	14	3.93	21.95	0.00
	Hybrids	27.23	12	2.27	20.06	0.00
	Between	1.88	1	1.88	1.68	0.20
	Error	8.20	55	0.15		
	Total	92.29	82			
TSSC/TA	Genotypes	553.55	27	20.50	4.18	0.00
	Parents	368.35	14	26.31	4.85	0.00
	Hybrids	185.05	12	15.42	3.59	0.00
	Between	0.15	1	0.15	0.01	0.91
	Error	245.14	50	4.90		
	Total	798.69	77			
