# Supplementary material for: Accelerating the Development of Heat Tolerant Tomato Hybrids through a Multi-Traits Evaluation of Parental Lines Combining Phenotypic and Genotypic Analysis
Source: Plants (Basel). 2021 Oct 13;10(10):2168. doi: 10.3390/plants10102168 (PMC8539001; doi:10.3390/plants10102168)
Supplement: Supplementary file 1 [file plants-10-02168-s001.zip › Table S8.pdf]

Table S8. Parental genotypes used in the crossing schemes for the constitution of F_1 hybrids. Experiment trials: C16, Campania 2016, C17, Campania 2017, C19, Campania 2019, P16, Puglia 2016, P17, Puglia 2017. Selected trait: HT, Heat-tolerance, Q, Quality.

Code	Origin	Common Name	Habitus	Experiment trials	Selected trait
E7	ITALY	Corbarino PC04	Indeterminate	C16, P16, C17, P17, C19	HT
E11	ITALY	Fabrizio	Determinate	C16, P16, C17, P17, C19	HT
E20	ITALY	Pizzutello	Indeterminate	C16, P16, C17, P17, C19	HT/Q
E36	ITALY	Seccagno	Indeterminate	C16, P16, C17, P17, C19	HT
E42	ITALY	PI15250	Determinate	C16, P16, C17, P17, C19	HT/Q
E45	ITALY	SM246	Determinate	C16, P16, C17, P17, C19	HT/Q
E48	ITALY	Vesuvio 2001	Indeterminate	C16, P16, C17, P17, C19	HT/Q
E55	SOUTH AMERICA	Latin american cultivar	Indeterminate	C16, P16, C17, P17, C19	HT/Q
E103	USA	Mini Red Current	Indeterminate	C16, P16, C19	Q
E109	EUROPE	V-L-101, Spain	Determinate	C16, P16, C17, P17, C19	HT
E111	USA	Black cherry	Indeterminate	C16, P16, C19	Q
LA2662	USA	Saladette	Determinate	C17, P17, C19	HT
LA3120	USA	Malintka 101	Determinate	C17, P17, C19	HT
PDLUC	ITALY	Lucariello	Indeterminate	C19	Q
PDVIT	ITALY	Cannellino Vitiello	Indeterminate	C17, P17, C19	HT/Q
