# Supplementary material for: Accelerating the Development of Heat Tolerant Tomato Hybrids through a Multi-Traits Evaluation of Parental Lines Combining Phenotypic and Genotypic Analysis
Source: Plants (Basel). 2021 Oct 13;10(10):2168. doi: 10.3390/plants10102168 (PMC8539001; doi:10.3390/plants10102168)
Supplement: Supplementary file 1 [file plants-10-02168-s001.zip › Table S9.pdf]

Table S9. List of molecular markers designed for each resistance gene. The primer sequences, restriction enzymes and references are also reported.

Gene	Marker	Primer pair	Enzyme	Reference
Mi-1.2	*Mi1.2_SCAR*	FW - TGGAAAAATGTTGAATTTCTTTTG<br>RV - GCATACTATATGGCTTGTTTAC	-	*Santos et al., 2020*
Ph-3	*Ph3_CAPS*	FW - TCGATCGTATGTAGACGATG<br>RV - AGGCAAATCTTGAAGAAGCA	*MspI*	*Jung et al. 2015*
Sw-5	*Sw5_dCAPS*	FW - GACGTTGTCCCAATTGTAGGC<br>RV - CCACTTCTTCAAGTCGAGTTG	*Hpy188I*	-
Tm-2	*Tm-2S_SCAR*	FW - TATCAACATGTCGCCCTGTGC<br>RV - AACTGTCTACCATAAAGCAGAC	-	-
Tm-2	*Tm-2R_SCAR*	FW - GTTATCAATATTTAGCCCTGTGC<br>RV - AACGGTCTACCGTAAAGTTGGC	-	-
Ty-3	*Ty-3_CAPS*	FW - TCTCAGGTGATGCTGAGCAC<br>RV - GAGAACGAAAACGAAATTTCAAAC	*RsaI*	*Kim et al. 2020*
Ve-1	*Ve-1_Nested*	FW - TTCTATCCCATATTTTCGACTG<br>RV - CATTGAGTTTATTGTTCCCAAG	-	-
Ve-1	*Ve-1_dCAPS*	FW - CAAGACTGGAGCTTTCTTACACT<br>RV - CGTACTCCAGCTGATTGAATG	*DdeI*	-
